# Tuning Pluronic Hydrogel Networks: Effects of Vancomycin Loading on Gelation, Rheological Properties, and Micellar Structures

**DOI:** 10.3390/gels11090688

**Published:** 2025-08-29

**Authors:** Michael J. Gaffney, Qi Han, Kate Fox, Nhiem Tran

**Affiliations:** 1 School of Science, STEM College, RMIT University, 124 La Trobe Street, Melbourne, VIC 3000, Australia; s3717834@student.rmit.edu.au; 2 School of Engineering, STEM College, RMIT University, 124 La Trobe Street, Melbourne, VIC 3000, Australia; kate.fox@rmit.edu.au

**Keywords:** hydrogels, Pluronic, F127, F108, F68, vancomycin, rheology, antibiotics, small-angle X-ray scattering

## Abstract

Thermoresponsive Pluronic hydrogels offer a promising platform for localised antibiotic delivery. However, how drug loading affects the structural integrity and gelation of these systems remains underexplored. This study evaluates the impact of vancomycin on the physicochemical and self-assembly behaviour of Pluronic F127, F108, and F68 hydrogels. Rheological analysis revealed that vancomycin altered the critical micellisation and gelation temperatures (CMT and CGT, respectively), accelerating gelation in weak gel systems but disrupting network formation in stronger gels. Small-angle X-ray scattering (SAXS) showed that vancomycin suppressed micellar ordering, particularly along FCC (111) planes in F127, without inducing a phase transition. Scanning electron microscopy (SEM) imaging confirmed reduced pore integrity in vancomycin-loaded F127 and F108 gels, while 35% F68 gels failed to form stable structures at the tested concentrations despite enhanced drug solubility. F127 (18%) and F108 (22–23%) maintained gelation at 37 °C with reasonable mechanical strength and partial cubic ordering, making them suitable candidates for drug-eluting gels. These findings inform the design of thermoresponsive hydrogels for localised, implant-associated antibiotic delivery.

## 1. Introduction

The current gold standard of local post-operative prophylactic treatment is antibiotic-loaded poly methyl methacrylate (PMMA) beads and has been established since the 1990s [1,2]. However, PMMA beads have drawbacks that make them less suitable for a modern treatment. PMMA beads have been reported to only release 25% of their loaded therapeutics, potentially causing concentrations to dip below the minimum bactericidal levels more quickly than required for long-term prophylaxis [3]. This can introduce the risk of biofilm formation around the implant, leading to chronic infection, sepsis, and implant rejection, all of which lead to revision surgeries and heightened demand on both the patient’s body and the hospital sector. PMMA’s non-degradable, bio-inert nature makes it a potential nidus for bacterial colonies [4,5,6] and can require revision surgeries for removal, which can cause further risk of infection and excessive strain on the patient by re-opening a wound [7,8]. Although PMMA beads are broadly accepted and do readily mix with a wide range of antibiotics [9], uncertainty surrounding potential inherent drawbacks could be remedied with a biodegradable replacement with more favourable drug release properties [10].

Hydrogels have emerged as a promising replacement for PMMA beads due to their biocompatibility, tuneable mechanical properties, and controlled-release capability for local drug encapsulation and delivery. Unlike PMMA, which typically releases over 90% of its antibiotic payload within the first 24–72 h, hydrogels have been reported to have superior sustained release for 7–14 days, offering improved efficacy against persistent surgical-site infections [11,12,13,14,15]. Furthermore, prophylaxis improves when antibiotics are loaded closer to the potential infection site, which, in the case of implants, is most commonly the tissue–implant interface [16]. Hydrogels applied directly onto implants ensure closer contact at the implant–tissue interface, whereas PMMA beads are typically loaded near the implants or surgical sites, reducing local antibiotic efficacy [4]. The physicochemical properties of the hydrogel-forming polymers strongly influence the injectability of the gel as well as drug retention and release, emphasising the need to characterise hydrogel-based systems for their suitability.

Poloxamer, known commercially as Pluronic, is a water-soluble triblock copolymer structured in an A-B-A structure of hydrophilic poly-ethylene oxide (PEO) blocks sandwiching a hydrophobic poly-propylene oxide (PPO) block. Although not biodegradable, Pluronic gel breaks down into unimers, which can be cleared by the kidneys, and is considered safe and non-toxic [17,18]. Pluronic hydrogels have been used as a pourable or injectable carrier for therapeutics due to their thermal gelation property, which allows them to be tuned to form a gel at body temperature while being a solution at room temperature [11]. At low temperatures, Pluronic exists as disordered unimers. Upon heating to the critical micellisation temperature (CMT), the dehydration of the PPO segments decreases their water solubility as they aggregate together to minimise interactions with water, forming a spherical hydrophobic micellar core ringed by a hydrophilic PEO corona [19,20]. Conversely, the concentration at which the micelles form at a given temperature is known as the critical micellisation concentration (CMC), with a lower CMC indicating a more stable micelle structure [21]. Further heating and dehydration lead to increased hydrophobicity in PEO and PPO blocks, driving unimer recruitment and denser packing of the PPO core, which influences the micelle size. Weak hydrogen bonds between micellar tails form, and hydrophobic interactions between micellar cores increase, both resulting in aggregation of micellar spheres and entanglement of PEO tails in the inter-micellar spaces. The resultant gelation of the micellar solution and formation of an ordered crystalline lattice structure at a given temperature is known as the critical gelation temperature (CGT). This sol–gel transition results in cubic micellar phases being formed, most notably more ordered face-centred cubic (FCC) structures for Pluronic F127 [22], and less ordered body-centred cubic (BCC) or hexagonal structures seen in F108 and F68 [23,24]. Cubic structures have different lattice spacing, micelle size, and channel geometry, all of which affect drug diffusion properties [25,26].

Pluronic types commonly researched for biomedical applications include F127, F108, and F68, which differ in PEO and PPO block size, i.e., F127 (PEO_100_PPO_65_PEO_100_), F108 (PEO_132_PPO_50_PEO_132_), and F68 (PEO_76_PPO_29_PEO_76_). A higher PPO content leads to a lower CMC and stronger PPO dehydration-driven gelation. As such F127 gels first at a given polymer concentration, followed by F108, and then F68. Therefore, an inverse relationship between PPO/PEO ratio and polymer concentration exists in Pluronic gel at room temperature. Phase diagrams are useful for understanding gel structure formation, function of temperature and polymer concentration; however, micelle behaviour is also sensitive to polymer purity, sample preparation, and solvent retention [27,28]. Furthermore, the addition of therapeutics has been shown to interfere with the hydrophobic interactions governing gelation, gel stiffness and elasticity, micellar gel structure, and phase response at body temperature [13,29,30]. These changes depend on both drug chemistry and concentration, with more hydrophobic therapeutics favouring micelle formation and aggregation, resulting in a lower CMT and CGT. Hydrophilic therapeutics generally have the reverse effect, increasing the CMT and CGT, and impeding the organisation of micelles into ordered cubic structures. Consequently, the quantity of therapeutics could impact the application of Pluronic gels for delivering prophylactics by impacting the stiffness and drug release rates of antibiotics from the gel [20,31,32,33,34,35,36]. More specifically, vancomycin has been encapsulated in F127 hydrogels in prior studies for the synergistic combination of an injectable gel and vancomycin’s ability to treat common post-operative infections [37,38]. However, despite the known effects of vancomycin and similar therapeutics on a gel’s physicochemical characteristics, no systematic comparison has been made between the characteristics (namely, gelation profile, micellar structure, and gel composition) of Pluronic F127, F108, and F68 when loaded with antibiotics such as vancomycin. Such a comparison is essential to determine whether Pluronic hydrogels can serve as effective, localised carriers for post-surgical antibiotic delivery to prevent infection, and to assess the comparative quality of different Pluronic types for withstanding the effects of vancomycin on the properties of the gel. 

This study aims to evaluate the effects of loading vancomycin—an antibiotic [39] commonly used to treat severe Gram-positive bacterial infections, including methicillin-resistant *Staphylococcus aureus* [40]—on the physicochemical properties of Pluronic F127, F108, and F68. Rheological assessments were performed to determine the influence of vancomycin on Pluronic gels’ CMT, CGT, and gelation viscosity. Small-angle X-ray scattering (SAXS) characterisation was performed to analyse the effects of vancomycin on the crystalline structures of Pluronic gels. We also investigated whether vancomycin disrupts micellar organisation and gel formation, thereby altering mechanical strength and drug incorporation behaviour. These findings aim to inform the formulation of thermoresponsive Pluronic gels for potential use in localised drug delivery systems.

## 2. Results and Discussion

### 2.1. Effects of Vancomycin on Pluronic Gelation and Solubilisation Behaviour

**Figure 1:**
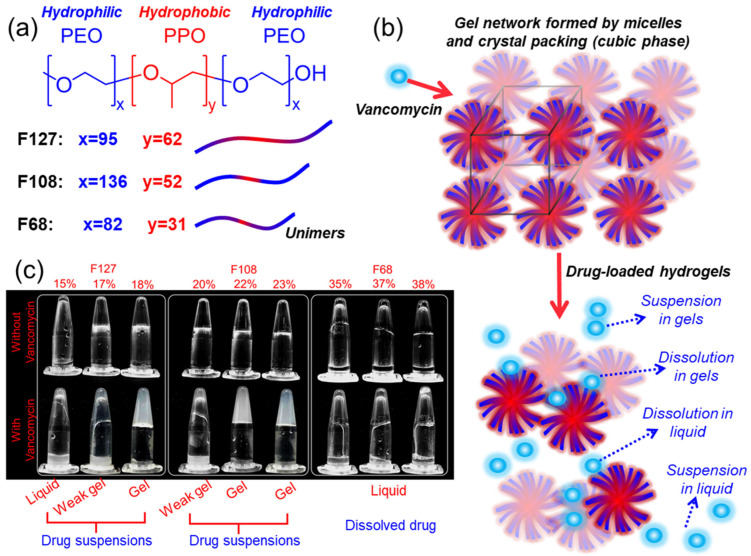
Composition, gelation, and drug–gel interaction outcomes of Pluronic hydrogels: (**a**) Structures of Pluronic F127, F108, and F68, showing differences in PEO/PPO block lengths. (**b**) Schematic of micelles packing into cubic gel networks and the effects of vancomycin loading. Drug incorporation leads to four outcomes: suspension in gels, dissolution in gels, dissolution in liquid, or suspension in liquid. (**c**) Tube inversion tests at 37 °C before and after vancomycin loading at various Pluronic concentrations. Vancomycin reduced the gel strength in some formulations (e.g., 17 wt% F127, 22 wt% F108), while F68 remained as a solution with full drug solubility.

The structural differences between Pluronic F127, F108, and F68, along with their block composition and hydrophilic–lipophilic balance (HLB), were determined by their PEO:PPO ratio, which in turn influenced micelle formation and gelation behaviour [20]. As shown in Figure 1a, F127 has the largest hydrophobic PPO block (y = 62), followed by F108 (y = 52) and F68 (y = 31). It has been shown that F127 has the lowest critical micellisation concentration (CMC) and the lowest gelation temperature [41]. Conversely, F68, with its smaller PPO block and higher hydrophilicity, requires higher concentrations to form gels [41]. In these three polymers, vancomycin loading is expected to alter the Pluronic temperature- and concentration-dependent gelation properties by interfering with micelle packing and micellar phase formation, inducing a less-ordered state (Figure 1b). To investigate this, tube inversion tests were performed along with rheology, SAXS, and structural analysis to assess the effects of vancomycin on gelation behaviour and micellar organisation.

To assess the gelation of hydrogels, 500 µL of neat and vancomycin-loaded Pluronic hydrogels at the listed concentrations (Table 1) was incubated in microcentrifuge tubes at 37 °C for 30 min, and then inverted and held for 1 min (Figure 1c). Neat 17 wt% and 18 wt% F127, along with all F108 gels, formed strong polymer networks that were resistant to flow, while 15 wt% F127 and all F68 samples remained fluid, indicating insufficient gelation under these conditions. Vancomycin-loaded Pluronic gels interfered with Pluronic gelation kinetics, as demonstrated by slightly more observable flow upon tube inversion than neat Pluronic at the same polymer concentration (Figure 1c). At 37 °C, only 18% F127 and 22% and 23% F108 retained strong enough gel structures to fully resist the inversion, while 17% F127 completely lost rigidity upon drug loading, suggesting the disruption of the hydrogel network. When loaded with vancomycin at higher than 1%, undissolved pockets of vancomycin were present at the bottom of the Eppendorf tube, identifiable by the shimmering light-reflective qualities of vancomycin, which indicated the suspension of the drug in the gel network. While F68 samples remained fluid, there were no visible drug precipitates, indicating better vancomycin dissolution than F127 and F108, possibly due to the higher hydrophilicity of the polymer. The same trends as for 37 °C were seen at 25 °C, as F127 and F108 formed weak gels, indicating disrupted networks compared to neat Pluronic, as well as visible vancomycin precipitates, which indicated limited drug compatibility (Figure 1c). In contrast, F68 exhibited less resistance to flow than the neat F68 samples but fully dissolved vancomycin, suggesting that its higher hydrophilicity favours drug solubility but weakens micelle packing and gelation at both 25 and 37 °C. These observations reflect the balance between gelation ability and drug compatibility, where strong gels may be more prone to drug precipitation, while more hydrophilic systems enhance solubility but with a poorer gel network.

### 2.2. Micellisation, Gelation, and Viscoelasticity of Pluronic and Pluronic–Vancomycin Hydrogels

#### 2.2.1. Pluronic Hydrogels

**Figure 2:**
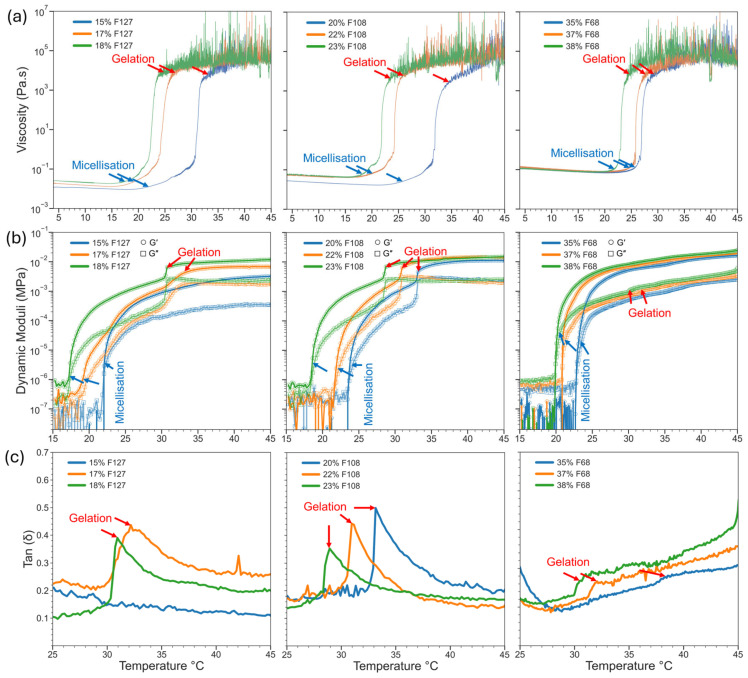
Rheological characterisation of Pluronic hydrogels at varying concentrations: (**a**) Viscosity profiles (flow mode) showing micellisation and sol–gel transitions. (**b**) Dynamic moduli (G′, G″) indicating micellisation onset and gelation plateaus. (**c**) Tan δ (G″/G′) highlighting gelation points (apex) and elastic behaviour (tan δ < 1). Gelation strengthened with higher polymer concentrations, notably in 17–18% F127, 22–23% F108, and 37–38% F68. Weak or no gelation was observed for 15% F127 and 35% F68.

The micellisation and gelation behaviour of neat F127, F108, and F68 hydrogels was assessed using temperature-dependent rheology in both flow and oscillatory modes (Figure 2a,b). The critical micellisation temperature (CMT) was defined as the point where viscosity increased and storage modulus (G′) split form the loss modulus (G″). From this point onwards, samples were characterised by a gel-like behaviour, as the elastic regime dominated the viscous regime. The critical gelation temperature (CGT) was marked by a sharp increase and then a plateau in dynamic viscosity. CGT values were confirmed by the peak of tan δ (i.e., G″/G′) in oscillation mode, which quantifies the balance between viscous and elastic behaviour. The tan δ peak and subsequent decrease indicated the final stages of micelle aggregation and strengthening of micelle–micelle interactions, along with the formation of a strong polymer network, as the elastic properties outweighed the viscous properties of the Pluronic hydrogel.

Based on the oscillatory modes shown in Figure 2b, all Pluronic solutions at low temperatures (below 20 °C) behaved as low-viscosity liquids. Upon heating, the transition from a micellised solution to a gel was observed as the dynamic moduli increased and G′ separated from G″, with minor shifts in recorded CMT between flow and oscillation modes due to different shear conditions (Table 2). Further heating led to finalisation of the sol–gel transitions, seen as sharp increases in viscosity and moduli and a plateau of the viscosity at 0.1 MPa. In this study, the CGT was not determined from the crossover temperature between G′ and G″ as commonly reported [42,43]. However, the recorded storage modulus values at the reported CGT aligned with previous works using Pluronic gels of a similar composition. The difference in the prolonged sol–gel transition is attributable to the measurements presented here being a continuous temperature ramp rather than only a few discrete points as previously studied [44]. The post-gelation plateau indicated an established linear viscoelastic regime and no loss in mechanical strength or ability to retain their shape upon further increases in temperature up to 45 degrees. Figure 2 demonstrated a prominent increase in dynamic moduli in 17% and 18% F127, 22% and 23% F108, and 37–38% F68, confirming robust gelation. In contrast, 15% F127 and 35% F68 showed no clear CGT or modulus increase, suggesting weak or absent polymer network formation at 37 °C.

Table 2 summarises the differences in the CMT, CGT, and tan δ of all samples. In general, higher Pluronic concentrations consistently lowered the CMT and CGT (arrows in Figure 2 and Table 2). This was attributed to greater PPO and polymer contents enhancing the hydrophobic interactions that govern gelation. Figure 2c shows that the tan δ values were below 1 across all gels, indicating predominantly elastic behaviour, although the higher tan δ values in weaker gels (e.g., 15% F127 and all F68 gels) indicated a concentration too low to have sufficient micellar aggregation to form a stable polymer network in the given temperature range. These findings aligned with the tube inversion tests (Figure 1), as samples with clear CGT and low tan δ values held their shape, while weaker samples remained a semi-viscous solution. These presented CGT values align with those previously reported for F127 [14], F108 [45], and F68 [24].

#### 2.2.2. Gelation and Viscoelasticity of Pluronic–Vancomycin Hydrogels

The same rheology experiments were performed with flow and oscillation temperature ramps for all samples to compare the effects of vancomycin on the micellisation and gelation temperatures and viscoelastic response. The vancomycin quantities were 10 mg, 25 mg, and 50 mg dissolved in 1 mL of water, which was used to dissolve Pluronic to make hydrogels, and the results from characterisation were compared with the neat drug-free results.

**Figure 3:**
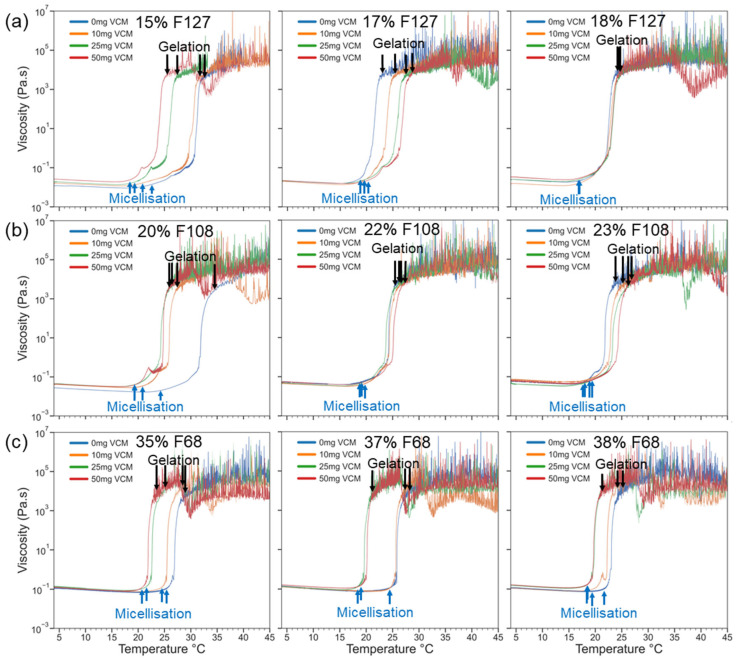
Viscosity vs. temperature profiles for Pluronic–vancomycin hydrogels of Pluronic F127 (**a**), F108 (**b**), and F68 (**c**). Critical micellisation and gelation temperatures (CMT and CGT) shift with increasing vancomycin loading. Stronger formulations (e.g., 18% F127, 22–23% F108) retain sharp transitions, while weaker ones show early gelation or loss of transition features. The blue arrows indicate CMT, and the black arrows indicate CGT.

Figure 3 shows the results of rheology measurements using the flow mode, which are sensitive to bulk viscosity changes and micelle alignment under continuous shear. Compared with the neat gels, increased vancomycin content led to sharper sol–gel transitions as well as micellisation and gelation at lower temperatures across all gels. This effect was more pronounced in the case of low polymer concentration and hydrophilic gels. The hydrophilic vancomycin induced a shift towards a more hydrophilic local microenvironment. This favoured the sol–gel transition of weak and F68 gels at lower temperatures, as evidenced by the CGT for 35% F68 ranging from 24 degrees with the 50 mg/mL vancomycin gel to 29 degrees with the neat 35% F68 gel. Similarly, 15% F127 and 20% F108 were drastically affected by the addition of vancomycin, with a 7-degree CGT difference in the former and a 9-degree difference in the latter. Contrastingly, 18% Pluronic F127 and 23% F108 gels were unaffected by vancomycin quantity. Across 0 mg/mL and 50 mg/mL samples, 18% F127 presented only a 1-degree CGT difference, and 23% F108 presented a 4-degree difference, both being much smaller CGT ranges compared with gels of a lower polymer concentration. Since both low-polymer-concentration gels and more hydrophilic gels presented a high CGT difference between the highest and lowest vancomycin quantities, it stands to reason that the polymer concentration and HLB of the Pluronic used both play a role in the degree to which thermal response is affected by vancomycin quantity. The stronger hydrophobic micelle interactions of lower-HLB gels appeared to act as a buffer against the disruption of the polymer network that vancomycin introduced in the higher-HLB gels. The hydrophobic process of PPO block dehydration was dominant over the local hydrophilicity that vancomycin introduced. This is corroborated by previous studies investigating higher polymer concentrations than the ones tested here, wherein vancomycin did not inhibit or interfere with the structural properties of Pluronic hydrogels, indicating that the concentrations tested here may reflect the turning point of vancomycin no longer affecting the structural properties of Pluronic [46,47,48].

**Figure 4:**
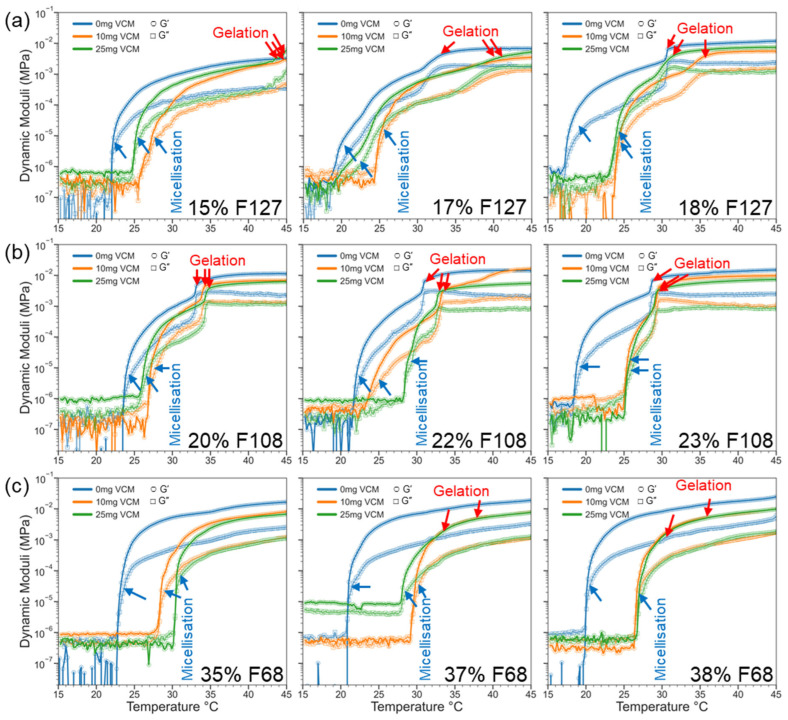
Oscillation-based temperature sweep with vancomycin-loaded Pluronic F127 (**a**), F108 (**b**), and F68 (**c**) under the same conditions as neat Pluronic. Storage modulus is denoted by circles with a line of best fit, and loss modulus is denoted by squares with a line of best fit. The blue arrow indicates CMT, and the red arrow indicates CGT.

**Figure 5:**
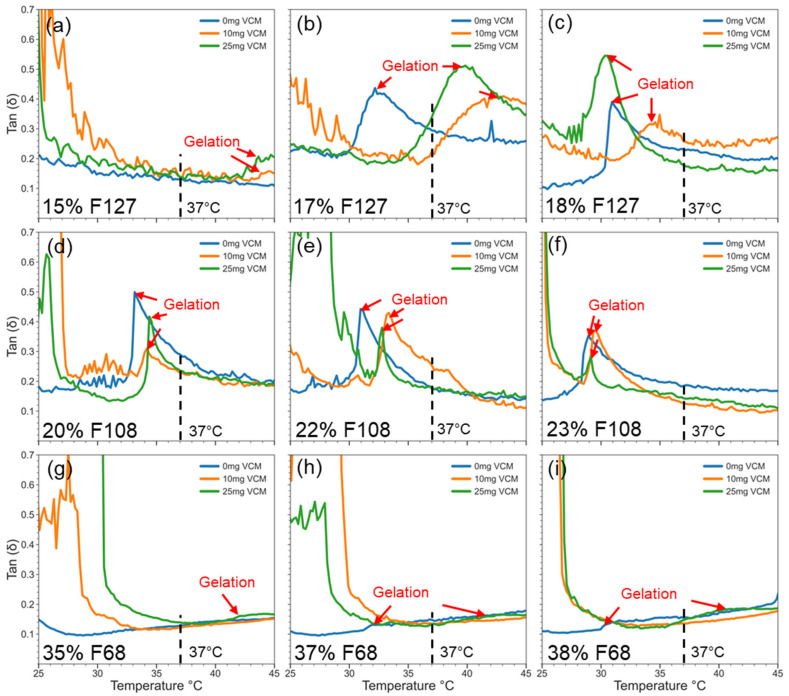
Tan δ (G″/G′) profiles across temperature sweeps of Pluronic F127 (**a**–**c**), F108 (**d**–**f**), and F68 (**g**–**i**). Micellisation is indicated by the lowering and plateauing of tan response, and gelation is indicated by the increase in tan response after the initial plateau. Gelation is marked by a tan δ peak (indicated by red arrows), which shifts with drug loading.

Unlike the flow mode, oscillatory measurements showed structural development of the micellar network under small deformations, with minimal shear-thinning influence on the CMT and CGT, allowing the system to develop purely from hydration-based thermal responses, and better reflecting the mechanical stiffness of the hydrogels. In flow mode, viscosity was measured as a function of temperature, while in oscillation mode, the dynamic moduli (G′ and G″) were measured instead, leading to differences in how gelation is represented in the respective plots. The results (Figure 4 and Table 3) showed that all gels showed a consistent increase in CMT and CGT when loaded with vancomycin. This stands in contrast to the flow temperature sweeps, wherein the weaker gels, including 15% F127 and all F68 samples (35%, 37%, and 38%), had a lower CMT and CGT with an increase in vancomycin concentration. For Pluronic F68 gels, gelation is driven predominantly driven by entanglement of long PEO chains and hydrogen bonding, which was assisted by the monodirectional movement of the Peltier plate in flow modes [47]. Oscillation-based temperature sweeps lack the mechanical assistance that the rotation of the Peltier plate provides for the PEO to assist in driving gelation, so the counterbalancing of the vancomycin’s hindrance of gelation was not present, resulting in a higher CMT and CGT. Stronger gels like 18% F127 and 22–23% F108 yielded comparable CGTs across both methods, with minimal drug impact, showing better structural resilience with higher polymer concentrations. The 17% F127 and 20% F108 gels displayed an increase in viscosity earlier in flow mode (improved micellisation), but Figure 5a,b show that the G′ and G″ transitions were delayed or broadened in oscillation (weaker gelation profile), with increased tan δ values in oscillatory mode (weaker polymer network). Among the polymers, F108 consistently showed the best agreement across flow and oscillation temperature sweeps, whereas F127 and F68 exhibited larger differences in CGT according to polymer concentration and vancomycin quantity. This suggests that vancomycin interfered with micelle packing and elastic network formation but the extent to which the vancomycin altered the gelation properties of the polymer was mitigated by the amphiphilic balance of F108 at the tested concentrations. F108 does not rely as heavily on PPO dehydration to gel (like F127), nor on the hydrogen bonding and tangling of long PEO chains (like F68), which may explain why the addition of vancomycin had a lesser effect on thermal response than for the other Pluronic variants. Similar results were presented for polymers with a stronger PPO dominance, such as 18% F127, where the effects of the vancomycin addition had a negligible impact on the thermic response. The level of vancomycin added was likely too small to act as a buffer to hinder the micellar aggregation in response to a temperature increase, likely due to the proximity and density of micelles in solution at higher concentrations. Both characteristics provide similar gelation kinetics upon the addition of vancomycin; however, the profile of the F108 dynamic moduli potentially indicates stronger suitability for practical applications due to the more robust and stable sol–gel transition when compared with F127. This could indicate that the Pluronic F108 will more predictably act as a fluid for mixing purposes at room temperature, more stably form gels at body temperature, and ensure a more consistent release of therapeutics from the hydrogel.

As summarised in Table 3, both flow and oscillatory modes showed similar CMT and CGT. However, it should be noted that vancomycin had a dual effect on CMT and CGT, depending on polymer strength. For weak gels like 15% F127 and all F68 samples, CMT and CGT decreased with increasing vancomycin, suggesting that the drug enhanced micelle formation, possibly by altering water–polymer interactions. In moderate-strength systems like 17% F127 and 20% F108, CGT increased slightly with vancomycin, likely due to steric or electrostatic interference with micelle aggregation. Conversely, strong gels such as 18% F127 and 22–23% F108 maintained stable gelation behaviour, with only minor shifts. This demonstrates that a well-developed polymer network can buffer against drug interference. Notably, F108 at 22–23% exhibited near-constant CMT and CGT values across all drug concentrations. This implies its suitability for drug-loaded formulations and stability in polymer networks despite the addition of vancomycin and the hindrance of micelle–micelle interactions. The F108 trend aligns with previous reports on a composite F68/F127 hydrogel, where tuning PPO and PEO balance to an HLB comparable with F108 improved gel stability and drug compatibility when compared with neat Pluronic hydrogels [32,49,50].

Figure 5 shows the tan δ (G″/G′) profiles, providing insights into the gels’ mechanical behaviour and drug interference. Across all samples, tan δ remained below 1, indicating elastic-dominant networks. In weak gels like 15% F127 and all tested F68 samples, vancomycin caused a modest rise in tan δ, often without a corresponding G′ increase. This result suggests micellisation without network strengthening. In moderate and strong gels, e.g., 17–18% F127 and 22–23% F108, tan δ shifted to higher temperatures with drug loading, matching the CGT delays seen in oscillatory data. Interestingly, the tan δ curves for F108 were less perturbed, indicating that its hydrophilic-rich structure may better accommodate vancomycin while maintaining network cohesion. Overall, the tan δ values confirm that while drug loading can delay or weaken gel formation in some systems, elasticity is retained post-gelation, especially in stronger formulations like F127 (18%) and F108 (22–23%). Since the F108 samples were both resistant to the hydrophilising effects of vancomycin in terms of maintaining gel hardness and maintaining similar CMT and CGT to neat F108, the polymer ratio of F108 may be ideal for facilitating vancomycin’s incorporation into the hydrogel matrix and preserving the physicochemical properties that make it suitable for in vivo use.

#### 2.2.3. Viscoelasticity of Pluronic–Vancomycin Hydrogels at 37 °C

To evaluate the mechanical integrity of Pluronic–vancomycin gels under physiological conditions, frequency sweep experiments were performed at a constant temperature of 37 °C, simulating in vivo conditions. This analysis provides insight into the gels’ resistance to deformation, network strength, and long-term mechanical stability under dynamic stress.

**Figure 6:**
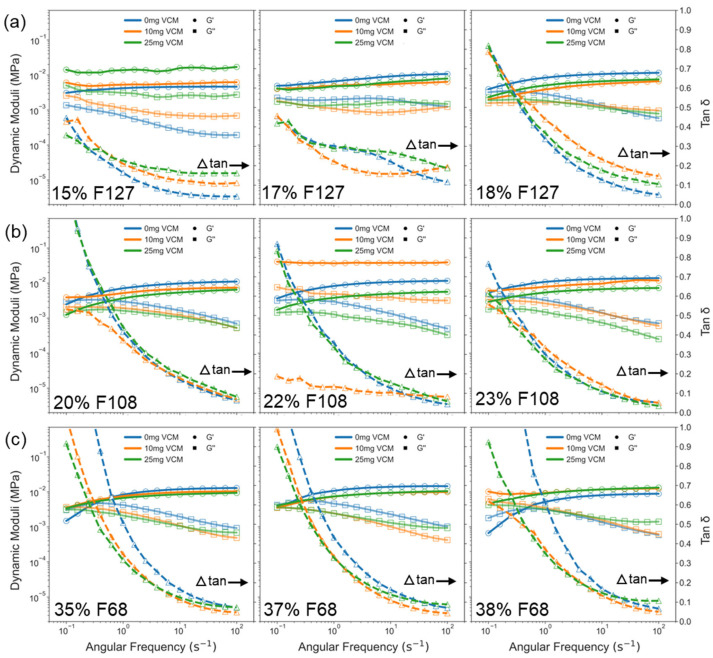
Frequency sweep analysis of vancomycin-loaded Pluronic F127 (**a**), F108 (**b**), and F68 (**c**) gels at 37 °C. Storage modulus (G′, circles), loss modulus (G″, squares), and tan δ (triangles) are plotted against angular frequency. F127 samples showed consistently strong elastic behaviour. F108 and F68 were more frequency-sensitive, especially at lower concentrations. All gels remained viscoelastic and stable across the tested range (0.1–100 rad/s).

The results show that all samples exhibited viscoelastic behaviour, with the storage modulus (G′) consistently higher than the loss modulus (G″) across most frequencies, confirming a gel-like behaviour (Figure 6). For F127 samples, G′ dominated G″ across the entire frequency range (0.1–100 rad/s), indicating strong and stable network structures. In F108 and F68, G′ and G″ were comparable at lower frequencies, with the storage and loss moduli overlapping. This was indicative of Pluronic exhibiting more of a viscoelastic than a gel response, but G′ eventually overtook G″ beyond ~0.5 rad/s to transition from a viscoelastic fluid to a gel. This difference from F127 reflects a characteristically more frequency-sensitive gel network in F108 and F68 samples, especially at lower concentrations (e.g., 20% F108, 35% F68). Across all three Pluronics, higher polymer concentrations slightly increased G′, but the effect was modest. This suggests that polymer concentration has limited influence on frequency-dependent behaviour once gelation is achieved. The tan δ (G″/G′) profiles revealed that F127 and F108 gels had the lowest tan δ values, followed by F68, aligning with prior gelation data (Figure 5). At 0.1 rad/s, the difference in tan δ between formulations was more distinct, indicating greater elastic character in F127, while F68 showed the highest tan δ, reflecting a more fluid-like response. However, at high frequencies (100 rad/s), the tan δ values across all gels converged to ~0.1, demonstrating that under rapid mechanical stress, all gels exhibit a similar deformation behaviour regardless of formulation. Importantly, no sample showed a decline in G′ with increasing frequency, confirming the network stability and absence of structural breakdown within the tested range. 

Overall, the frequency sweep results indicate that Pluronic F127 and F108 gels, particularly at 17–18% and 22–23%, respectively, maintain stronger and more elastic networks at 37 °C, while F68 gels remain weaker and more sensitive to frequency. These findings further support the selection of F127 and F108 systems for drug delivery, as they combine sufficient gel strength, frequency stability, and elastic response suitable for physiological environments.

### 2.3. Microstructural Morphology by SEM

**Figure 7:**
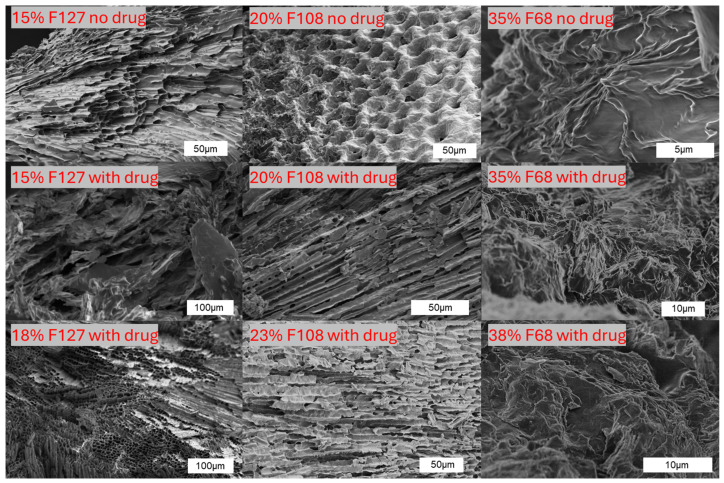
SEM images of freeze-dried Pluronic hydrogels with and without vancomycin. Neat gels (top row) show distinct morphologies for F127, F108, and F68. Drug-loaded samples (bottom rows) exhibit denser, less porous structures, especially at higher concentrations (e.g., 18% F127, 23% F108), consistent with gelation and viscoelastic data.

To further assess the physical network of the hydrogels, SEM imaging was performed on freeze-dried Pluronic–vancomycin samples (Figure 7). The surface morphologies revealed clear differences in pore orientation, density, and compactness among different Pluronic types and drug-loading conditions. The neat Pluronic hydrogels displayed distinct structures: 15% F127 showed layered lamellae, 20% F108 exhibited a honeycomb-like porous structure, and 35% F68 had sparse fibrous arrangements. Upon vancomycin incorporation, the microstructure became more compact and less porous in most samples, especially in F127 and F108. This indicates drug-induced densification or collapse of the gel matrix. This is consistent with the reduction in gel stiffness and increased tan δ observed in earlier rheology sections. The 15% F127 gel with drug lost the ordered lamellar architecture, becoming fragmented and disordered. In contrast, 18% F127 and 23% F108, which retained good gelation profiles, also maintained more coherent structures with aligned or layered features. F68 gels, both with and without vancomycin, displayed a loosely packed, amorphous irregular network across all concentrations, matching their weak rheological performance.

These morphological features reflect the gels’ internal architecture and validate the rheological findings, weaker gels show disrupted, p0-open networks, while stronger gels maintain tighter, organised microstructures, supporting their mechanical robustness at 37 °C.

### 2.4. SAXS Characterisation of Hydrogel Structure

#### 2.4.1. Pluronic Hydrogels: Phase Formation with Temperature and Concentration

To identify the formation of crystalline micelle phases, SAXS was used to investigate the internal ordering of Pluronic hydrogels across increasing temperatures. Phase behaviour in Pluronic systems is known to depend not only on polymer concentration and temperature but also on diblock content. This may alter whether face-centred cubic (FCC), body-centred cubic (BCC), or hexagonal (Hex) structures are formed [51].

**Figure 8:**
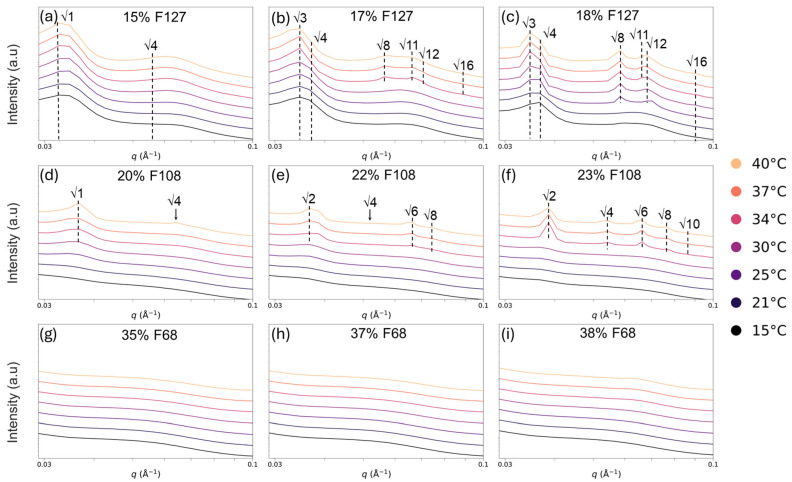
SAXS profiles of neat Pluronic hydrogels of F127 (**a**–**c**), F108 (**d**–**f**), and F68 (**g**–**i**) at various temperatures (15–40 °C). Ordered FCC or BCC phases form only at medium and high concentrations of F127 and F108. Increasing temperature enhances long-range ordering, while low-concentration and all F68 samples remain disordered.

At lower polymer concentrations (15% F127, 20% F108, and all F68 samples), no obvious SAXS peaks were observed (Figure 8). This indicates a lack of long-range order in the gel. This is consistent with rheology and SEM results showing weak or no gelation in these systems. In contrast, medium and high concentrations of F127 (17% and 18%) and F108 (22% and 23%) exhibited distinct Bragg peaks corresponding to an FCC structure with ratios of √3, √4, √8, √11 or a BCC structure with ratios of √2, √4, √6, √8, which intensified with increasing temperature from 25 °C to 40 °C (Figure 8). Figure 8b demonstrates that large primary peaks at √3 were the first to form in the F127 gel, indicating that the micelle arrangement was primarily dictated by the nearest-neighbour interactions rather than long-range order. Increasing temperatures resulted in a decrease in the primary peak intensity attributed to the micellar reorganisation into a system less defined by nearest-neighbour interactions and more defined by periodic FCC and BCC structures. This assessment was also made due to the secondary and tertiary peaks in the FCC and BCC systems becoming broader while maintaining their distinct peaks from 34 to 40 °C. F127 transitioned from a disordered to an FCC structure with increasing temperature and concentration, with clear √3, √4, √8, and √11 peaks appearing (Figure 8). Similarly, 23% F108 formed BCC structures at higher temperatures, as evidenced by the √2, √4, √6, and √8 peaks (Figure 8f). Notably, the onset of crystallisation occurred near or slightly above the CGT, i.e., 30 °C, as reported in earlier rheological analyses (Table 3). This suggests that crystal formation is a thermally driven refinement of the existing gel network. At higher temperatures (over 34 °C), the primary peak intensities decreased, while the secondary peaks broadened, indicating micellar reorganisation toward more dynamic but ordered states. All F68 samples (35–38%) remained structurally disordered (Figure 8g–i), despite forming weak gels by rheology. Reports in the literature indicate that Pluronic F68 requires >45% to form a BCC phase at 35 °C; thus, the concentrations used here are below the threshold for self-assembly into lattice structures [24]. Table 4 summarises this temperature–concentration dependence: for example, FCC structures only emerge in 17% and 18% F127 at ≥35 °C, and BCC-like ordering appears in 23% F108 only above 35 °C. This concentration threshold highlights that a critical level of micelle density and dehydration is required for cubic lattice assembly, consistent with the gelation behaviour.

#### 2.4.2. Micellar Organisation of Pluronic–Vancomycin Hydrogels at 37 °C

**Figure 9:**
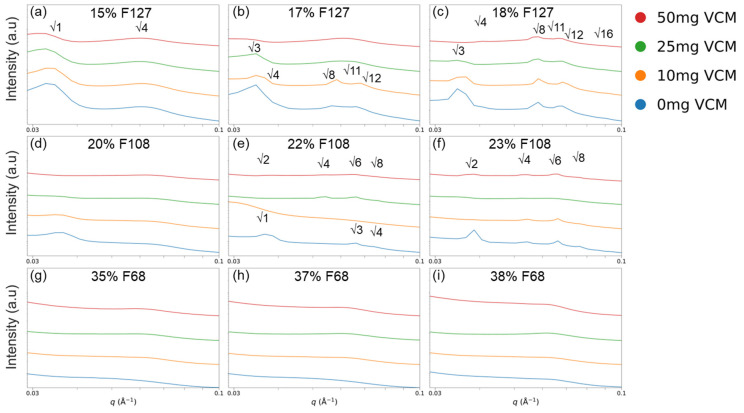
SAXS profiles of vancomycin-loaded Pluronic hydrogels of F127 (**a**–**c**), F108 (**d**–**f**), and F68 (**g**–**i**) at 37 °C. Higher vancomycin concentrations reduced the intensity and number of diffraction peaks, especially the primary peak, reflecting disruption of nearest-neighbour micelle packing. No new phase transitions were observed.

To assess the structural impact of drug loading, SAXS was performed at 37 °C on vancomycin-loaded Pluronic hydrogels across increasing drug concentrations (Figure 9). In general, the presence of vancomycin suppressed both the intensity and clarity of the SAXS peaks, particularly the primary peak that is associated with nearest-neighbour micelle interactions. The results are summarised in Table 5.

As shown in Figure 9, 17% and 18% F127, as well as 22% and 23% F108, formed detectable FCC or BCC phases with drug present. All other hydrogels failed to form cubic structures and had no discernible peaks. Vancomycin loading appeared to reduce the micellar organisation in all samples that formed cubic structures with a neat gel, from ordered FCC or BCC structures to less-ordered structures, as evidenced by the reduced peak intensity. The 17% F127 and 22% F108 gels lost detectable crystalline phases at higher drug concentrations (Figure 9b,e), highlighting the destabilising effect of vancomycin on lattice formation in previously ordered gels. The F108 gels at the tested concentrations previously showed the most stable rheological performance and the most defined SEM structures; however, increasing vancomycin concentrations (10, 25, and 50 mg/mL) progressively decreased the long-range order. At 50 mg/mL vancomycin, several FCC peaks (e.g., √3 in 17% F127) were no longer observable, while secondary peaks were broadened or missing, suggesting interference in periodic micelle alignment. The effect that vancomycin had on peaks beyond the first was less pronounced than the suppression effect that it had on the primary peak. The suppression of these peaks implies that vancomycin affects the ability of micelles to organise into well-defined crystalline lattices.

**Figure 10:**
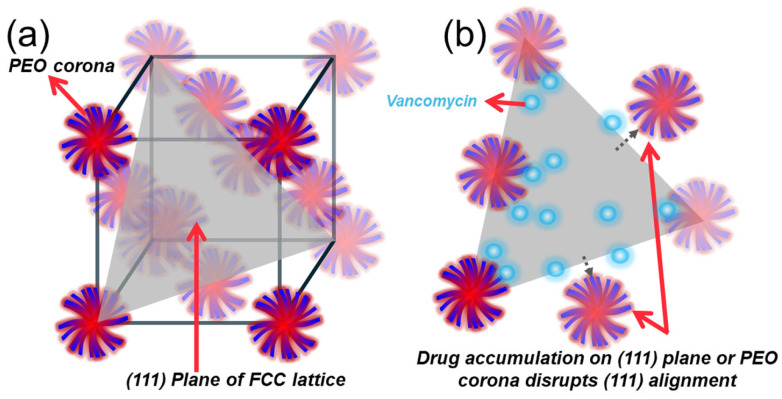
(**a**) Schematic of micellar arrangement in an FCC lattice, highlighting the (111) crystallographic plane formed through tight corona–corona overlap of Pluronic micelles. (**b**) Disruption of (111) alignment upon vancomycin loading. Drug molecules (blue) accumulate in inter-micellar regions or PEO coronas, interfering with nearest-neighbour packing. This localised interference weakens short-range order, resulting in the suppression of SAXS √3 reflections without inducing a full phase transition.

The disruption of the primary SAXS peak—for example, the √3 reflection in F127 (Figure 9c)—suggests that vancomycin interferes with nearest-neighbour micelle packing, likely through steric hindrance, hydration changes, or electrostatic repulsion at the corona level. In an FCC lattice, this √3 peak corresponds to the (111) crystallographic plane—the most densely packed direction. The selective loss of this peak at high vancomycin concentrations (notably in 18% F127 with 50 mg/mL vancomycin) indicates preferential disruption along these planes. This supports the hypothesis that vancomycin molecules are localised in inter-micellar regions, particularly in (111) planes where corona–corona overlap is maximal—either occupying interstitial water or interacting with PEO chains. This mechanism is illustrated in Figure 10, where vancomycin accumulation along the (111) direction weakens the micellar alignment and scattering contrast. The absence of new peaks (e.g., √2, √4, √6) indicates that the FCC mesophase is maintained, which contrasts with the transition to a less-ordered BCC that was seen in previous studies [29,52,53]. While 18% F127 at the highest tested vancomycin concentration appears BCC-like due to the primary peak suppression, this likely reflects selective peak suppression rather than a structural transformation. Prior studies displayed similar effects, wherein the effects of adding ibuprofen to Pluronic solutions increased the degree of micellar order and lowered gelling temperature due to being a hydrophobic compound, whereas hydrophilic ibuprofen sodium increased the gelling point of Pluronic F108, highlighting the effect that the hydrophobicity of added therapeutics has on the micellar ordering and gelation profiles of Pluronic gels [54,55]. This result aligns with the Hofmeister series, as the ionic effects of the hydrophilic vancomycin on the hydrogel would push the gelling of Pluronic systems to higher temperatures due to the kosmotropic effects that it has on the hydrogel matrix [56].

F127 samples showed undissolved vancomycin (Figure 1c), which further supports poor compatibility with the PPO-rich core. The SAXS data is consistent with these findings, showing disruption localised to hydrophilic corona regions. These structural disruptions align with rheological results showing increased tan δ and reduced G′, reinforcing that vancomycin interferes with micelle–micelle interactions without reorganising the overall lattice.

F108 retains more structural order even with the drug present (Figure 9f), suggesting better solubilisation due to its longer PEO chains and higher HLB. This supports the idea that drug compatibility varies with Pluronic type: in F127, vancomycin is less soluble and accumulates locally along packed micellar planes, while in F108 it disperses more uniformly in the corona region without severely disrupting lattice formation.

These findings are supported by the rheological data, where CGT was delayed and storage moduli weakened, and by SEM imaging, which showed collapsed or less porous networks in vancomycin-loaded gels. Together, these results confirm that vancomycin disrupts micelle–micelle spacing and suppresses structural ordering, particularly in FCC-type F127 and BCC/Hex-like F108 systems. These disruptions likely occur in the inter-micellar aqueous phase, where drug molecules interfere with corona overlap and hinder the regular packing needed for lattice formation.

## 3. Conclusions

This study comprehensively evaluated the impact of vancomycin loading on the thermoresponsive gelation, mechanical behaviour, and microstructural ordering of Pluronic F127, F108, and F68 hydrogels. Across all systems, vancomycin was shown to interfere with micellar interactions, but the degree and nature of interference varied based on polymer type and concentration. At low polymer concentrations (e.g., 15% F127, 20% F108, 35% F68), vancomycin accelerated gelation slightly, likely due to early dehydration and micelle formation promoted by localised osmotic effects. However, these systems lacked sufficient network strength, as shown by their weak rheological properties, poor structural ordering in SAXS, and incomplete gelation at 37 °C. Thus, they are unsuitable for in vivo drug delivery, where mechanical robustness is essential. In intermediate-to-high concentrations (17–18% F127, 22–23% F108), the opposite trend was observed: drug loading delayed micellisation and gelation, increased tan δ, and suppressed elastic moduli. SAXS revealed that vancomycin selectively disrupted micelle packing—especially nearest-neighbour interactions along the (111) FCC planes in F127—without inducing phase transitions. These disruptions were attributed to drug accumulation in inter-micellar water or PEO coronas, impeding corona–corona overlap and weakening crystalline lattice formation. Nonetheless, F108 (22–23%) and F127 (18%) still retained sufficient ordering, viscoelastic stability, and gelation at body temperature, making them promising candidates for controlled drug delivery. These conclusions are supported by SEM imaging, which revealed more porous and structured morphologies in these formulations, and by tube inversion tests that confirmed their ability to maintain gel strength at 37 °C. Pluronic F68 was consistently unable to form stable gels or crystalline phases across all conditions, even with the drug present, due to its high hydrophilic–lipophilic balance and shorter PPO block, rendering it unsuitable for further study in this context. These findings highlight the dual role of vancomycin: it can either promote or disrupt gelation, depending on the polymer network strength. For biomedical applications, this underscores the need to match drug loading with polymer formulation to maintain desirable gelation kinetics and mechanical strength. Additionally, the suppression of ordered cubic phases by drug loading has direct implications for diffusion and release kinetics, which may become less predictable in disrupted networks. To optimise these systems, additives such as kosmotropic salts or hydrophobic modifiers could be employed to strengthen PPO-driven micellisation, reduce water content in the corona region, and mitigate vancomycin’s disruptive effect on the (111) plane. This would help maintain gelation properties while enhancing drug encapsulation and retention. Overall, F108 (22–23%) and F127 (18%) emerge as the most viable formulations for sustained local delivery of antibiotics, offering a balance of thermoresponsive gelation, mechanical integrity, and structural organisation, which are critical for post-operative or implant-associated drug release platforms.

## 4. Materials and Methods

### 4.1. Pluronic Hydrogel Preparation

Pluronic F127, F108, and F68 (Sigma Aldrich, St. Louis, MO, USA) were dissolved in Milli-Q water at defined concentrations (Table 1) and stored at 4 °C. F127 hydrogels were prepared at 15%, 17%, and 18% wt; F108 at 20%, 22%, and 23% wt; and F68 at 35%, 37%, and 38% wt. These concentrations have been used previously, since they transition from a solution at room temperature to a gel at body temperature [14,57].

For vancomycin-loaded hydrogels, vancomycin hydrochloride powder was weighed and dissolved in Milli-Q water to form three stock solutions with increasing concentrations of 10, 25, and 50 mg/mL. These were vortexed until full vancomycin dissolution. Pluronic was weighed out to reach the same polymer concentrations as before, and then dissolved in the vancomycin-loaded water instead of pure Milli-Q water to obtain a drug-loaded solution with the same polymer concentrations as the neat counterparts. This resulted in the same Pluronic polymer concentrations, with three incremental vancomycin concentrations to test the degree of drug-induced changes in the gel properties. Pluronic–vancomycin solutions were vortexed and homogenised, and then stored at 4 °C prior to characterisation.

### 4.2. Rheometric Experiments and Analysis

Rheological measurements were performed with a rheometer (Discovery HR-3, TA Instruments, New Castle, DE, USA), following a modified method from Han et al. [58]. The samples were equilibrated at 25 °C for 10 min before being placed in a 20.0 mm, 1° stainless steel Peltier plate geometry with a 50 µm gap.

Flow-based temperature ramp experiments were performed over the temperature range of 4–45 °C at a constant shear rate of 0.1 s^−1^. After a 300 s equilibration time at 4 °C, the temperature was increased at a rate of 1 °C per minute. The stress, shear rate, viscosity, step time, temperature, and normal stress were recorded, and the results were plotted as viscosity (Pa.s) vs step time (s).

Oscillatory temperature ramps and frequency sweeps were performed consecutively. For the temperature ramp, the samples were soaked for 180 s at 15 °C before temperature ramping from 15 to 45 °C at 2 °C per minute, at a 0.5% constant strain and a set frequency of 1 Hz. The outputs for the temperature ramp were storage modulus (G′), loss modulus (G″), and tan delta (δ) (the ratio of G″/G′, with lower values corresponding to a harder gel). G′ and G″ are also referred to as the elastic and viscous modulus, respectively, since they reflect the material’s elastic and viscous behaviour. After finalisation of the temperature ramp, the temperature was reduced to 37 °C and maintained for 180 s to ensure stable thermal response at homeostatic temperatures. Frequency sweeps commenced from 0.1 rad/s to 100 rad/s, with a constant 1% strain. The outputs were elastic modulus (G′), viscous modulus (G″), and tan delta (δ) as a function of angular frequency (ω).

### 4.3. Scanning Electron Microscopy (SEM)

The microstructure of lyophilised Pluronic hydrogels with and without vancomycin was examined using a field-emission scanning electron microscope (FEI Verios 460L XHR-SEM, Thermo Fisher, Hillsboro, OR, USA). Hydrogel samples were prepared with and without 10 mg of vancomycin dissolved in 1 mL of water, corresponding to loaded samples of 15% F127, 20% F108, and 35% F68 (Table 1). Samples were snap-frozen in liquid nitrogen and lyophilised for 48 h to preserve structural integrity. Representative micrographs were collected to compare the surface morphology and internal architecture across different Pluronic types and vancomycin concentrations. Structural changes were qualitatively assessed based on porosity, layer compactness, and network continuity.

### 4.4. SAXS Experiments and Analysis 

SAXS characterisation was performed at the Australian Synchrotron, Melbourne, in the SAXS/WAXS beamline. The samples were characterised with a beam energy of 11.5 keV with a camera length of 1012 mm; 100 µL of each gel listed in Table 1 was transferred to a 96-well plate 3 days prior and stored at 4 °C until testing. SAXS data was collected at various temperatures from 15 °C to 40 °C, in 5 °C increments, with an additional measurement at 37 °C to simulate body temperature. The samples were allowed to sit for 3 min at each temperature to establish thermal equilibrium before the scattering data was collected. SAXS data was processed using Scatterbrain software (V1.230) for peak identification and fitting Bragg peaks to known crystal structures. D-spacing was calculated based on the peak positions to assess micelle packing and structure [59,60].

## Figures and Tables

**Table 1:** The composition of Pluronic (F127, F108, and F68) and vancomycin in hydrogels.

	Polymer in 1 mL of Water (mg)	Polymer Concentration (wt%)	Vancomycin Quantity in 1 mL of Water ^a^		
			10 mg	25 mg	50 mg
			wt%	wt%	wt%
F127	175	15%	0.8%	2.1%	4.1%
	200	17%	0.8%	2.0%	4.0%
	225	18%	0.8%	2.0%	3.9%
F108	250	20%	0.8%	2.0%	3.8%
	275	22%	0.8%	1.9%	3.8%
	300	23%	0.8%	1.9%	3.7%
F68	550	35%	0.6%	1.6%	3.1%
	575	37%	0.6%	1.6%	3.1%
	600	38%	0.6%	1.5%	3.0%

^a^: Vancomycin quantities were standardised by weight (10, 25, or 50 mg per 1 mL of hydrogel), ensuring that each hydrogel formulation contained the same absolute amount of drug across different Pluronic types.

**Table 2:** Micellisation and gelation behaviour of Pluronic measured using flow and oscillation modes.

Polymer	Concentration (wt%)	CMT from Flow/Osc (°C) ^a^	CGT from Flow/Osc (°C) ^b^	Tan δ at 37 °C ^c^	Rheology Summary
F127	15%	22/22	33/-	<0.6	Weak incomplete gelation ^d^
	17%	19/19	27/33	0.32	Good gelation, elastic plateau
	18%	17/17.5	25/31	0.25	Strong gel, early gelation
F108	20%	26/24	35/33	0.45	Gradual gelation, moderate strength
	22%	20/22	26/31	0.29	Strong gel, steady plateau
	23%	18/19	24/29	0.27	Strong gel, stable LVR
F68	35%	26/23.5	30/-	<0.6	Solution state, no clear CGT
	37%	25/21.5	28/32	0.34	Weak gel, low-strength network
	38%	22/20.5	26/30.5	0.3	Weak but structured gel

^a^: CMT was measured by the point where viscosity increased by the flow mode. ^b^: CGT was determined by G″ split form G′. ^c^: Tan δ values below 1 are defined as gels, while high δ values (˂0.3) are related to the formation of stronger gels (elastic, little viscous loss). ^d^: Weak gelation was visualised with no resistance to flow when inverted, good gelation as a slight fluid movement when inverted, and strong gelation as complete resistance to flow when inverted.

**Table 3:** Micellisation and gelation behaviour of vancomycin-loaded Pluronic hydrogels measured using flow, and tan response measured using oscillation modes at varying temperatures.

Polymer Conc. (wt%)	CMT (°C) of Samples with 0/10/25 mg Vancomycin	CGT (°C) of Samples with 0/10/25 mg Vancomycin	Tan δ at 37 °C (25 mg Vancomycin)	Rheology Summary
F127 15%	22/21/19	33/32/27.5	>0.6	Weak gel, improved by vancomycin
F127 17%	19.0/19.5/19.5	24/25.5/27.5	0.37	CGT delayed with vancomycin, moderate strength
F127 18%	17.0/17.0/17.0	24/24.5/24.5	0.29	Strong, stable gelation
F108 20%	26/21/19	35/27.5/26.5	0.35	CMT/CGT reduced by vancomycin, moderate gel
F108 22%	20/22/22	26/26.5/26.5	0.27	Very stable gelation, low interference
F108 23%	18/19/18	24/25/26	0.25	Strong gel, minimal change
F68 35%	26/25/22	30/28.5/25	>0.5	Weak gel, early gelation with vancomycin
F68 37%	25/25/19	28/27/21	0.33	Gelation improved, but low strength
F68 38%	22/20/19	26/24/21	0.3	Early CGT, still weak gel

**Table 4:** Phase behaviour of F127, F108, and F68 at various concentrations and temperatures.

Temp (°C)	F127-15%	F127-17%	F127-18%	F108-20%	F108-22%	F108-23%	F68-35%	F68-37%	F68-38%
15	Less ordered	Less ordered	Less ordered	Solution	Solution	Solution	Solution	Solution	Solution
20	Less ordered	Less ordered	FCC (311 *)	Solution	Solution	Solution	Solution	Solution	Solution
25	Less ordered	Less ordered	FCC (307)	Less ordered	Less ordered	Less ordered	Solution	Solution	Solution
30	Less ordered	Less ordered	FCC (308)	Less ordered	Less ordered	Less ordered	Solution	Solution	Solution
35	Less ordered	FCC (318)	FCC (309)	Less ordered	Hex (171)	BCC (237)	Solution	Solution	Solution
37	Less ordered	FCC (319)	FCC (310)	Less ordered	Hex (170)	BCC (234)	Solution	Solution	Solution
40	Less ordered	FCC (316)	FCC (318)	Hex (172)	BCC (240)	BCC (241)	Solution	Solution	Solution

*: The brackets indicate the D-spacing obtained from SAXS patterns.

**Table 5:** Vancomycin–Pluronic phase structures at 37 °C.

Temp (°C)	F127-15%	F127-17%	F127-18%	F108-20%	F108-22%	F108-23%	F68-35%	F68-37%	F68-38%
0 mg	Less Ordered	FCC	FCC	Less Ordered	Hex	BCC	Less Ordered	Less Ordered	Less Ordered
10 mg	Less Ordered	FCC	FCC	Less Ordered	BCC	BCC	Less Ordered	Less Ordered	Less Ordered
25 mg	Less Ordered	Less Ordered	FCC	Less Ordered	Less Ordered	BCC	Less Ordered	Less Ordered	Less Ordered
50 mg	Less Ordered	Less Ordered	FCC	Less Ordered	BCC	BCC	Less Ordered	Less Ordered	Less Ordered

## Data Availability

The raw data supporting the conclusions of this article will be made available by the authors on request.
